# YOLOv11-SR: An Enhanced YOLOv11-Based Framework for Defect Detection in Complex Substation Inspection Images

**DOI:** 10.3390/s26165010

**Published:** 2026-08-07

**Authors:** Xitong Liu, Jianwen Hu, Zaiying Jiang, Leite Zeng, Yanhui Xi

**Affiliations:** 1 School of Electrical & Information Engineering, Changsha University of Science and Technology, Changsha 410114, China; 2 School of Artificial Intelligence, Changsha University of Science and Technology, Changsha 410114, China; hujianwen@csust.edu.cn (J.H.);

**Keywords:** substation inspection, explicit fault detection, YOLOv11-SR, detail preservation, adaptive feature fusion

## Abstract

Accurate detection of explicit faults in substation equipment is critical for intelligent substation inspection. However, practical inspection scenes often involve small objects, blurred boundaries, background clutter, occlusion, and large-scale variations, which significantly challenge the reliability of existing object detection methods. To address these issues, this paper proposes YOLOv11-SR, an improved object detection framework for explicit fault detection in complex substation environments. Specifically, a Shallow Detail Preservation Attention Module (SDPAM) is introduced to preserve fine-grained shallow details during downsampling, while a Position-Relation Guided Adaptive Fusion Module (PRGAFM) is designed to enhance adaptive multi-scale feature fusion under cluttered backgrounds. Experimental results show that YOLOv11-SR achieves 89.9% Precision, 87.8% Recall, 89.0% F1-score, 90.6% mAP@0.5, and 64.8% mAP@0.5:0.95, outperforming representative detectors including YOLOv5, YOLOv7, YOLOv11, YOLOv13, and YOLO-SS-Large. Category-level analysis and ablation studies further verify the effectiveness of the proposed method, particularly for detecting small and blurred targets in complex scenes. These results demonstrate the effectiveness and robustness of YOLOv11-SR for intelligent substation defect inspection.

## 1. Introduction

Automatic defect detection in substation inspection images is essential for ensuring the reliability and safety of power grids. With the increasing deployment of intelligent inspection platforms, such as UAVs (Unmanned Aerial Vehicles) and robotic vision systems, deep learning-based visual detection has become an indispensable technique for reducing inspection costs and improving operational efficiency. However, substation inspection images are usually acquired under long-distance and complex environmental conditions, where defect targets often appear small, weakly textured, and blurred. In addition, cluttered backgrounds, illumination variation, and occlusion further increase the difficulty of accurate localization and classification [1,2,3].

Recent advances in deep learning-based object detection have substantially improved visual inspection performance. In particular, one-stage detectors represented by the YOLO family have evolved from YOLO [4] to YOLOv7 [5], YOLOv11 [6], and YOLOv13 [7], and have been widely adopted in practical inspection tasks because of their favorable trade-off between detection accuracy and inference speed. Meanwhile, multi-scale feature learning and feature enhancement have been widely recognized as effective strategies for improving the representation of weak and small targets in complex scenes [8,9]. These observations are highly relevant to substation inspection, where defect categories often exhibit strong scale variation and subtle defect details are easily submerged in cluttered backgrounds.

Despite these advances, several limitations remain in existing studies on power-equipment inspection. Many methods focus on specific equipment categories or single defect types, while unified detection for multiple defect classes in complex substation scenes remains insufficient. Moreover, the balance among detection accuracy, inference speed, and practical applicability has not been fully resolved. To address these issues, this paper proposes YOLOv11-SR (You Only Look Once version 11 with Shallow-detail and Relation-aware modules), an enhanced detection framework for complex substation inspection images. By introducing a shallow-detail enhancement module and a relation-aware feature fusion module, the proposed method improves the detection of small and blurred defects under cluttered substation backgrounds.

The main contributions of this work are summarized as follows:

(1) A novel defect detection framework, YOLOv11-SR, is proposed for complex substation inspection images, which jointly improves shallow-detail representation and relation-aware feature fusion.
(2) A Shallow Detail Preservation Attention Module (SDPAM) is designed to alleviate shallow information loss during downsampling, thereby enhancing the detectability of small and blurred defects.
(3) A Position-Relation Guided Adaptive Fusion Module (PRGAFM) is introduced to adaptively fuse multi-scale features according to spatial and relational cues, leading to improved feature integration in complex scenes.
(4) Extensive experiments demonstrate that the proposed method achieves superior detection performance over baseline detectors while maintaining practical inference efficiency.

The remainder of this paper is organized as follows. Section 2 reviews the related work. Section 3 presents the overall architecture of YOLOv11-SR, together with the proposed SDPAM and PRGAFM modules. Section 4 describes the experimental settings and comparative results. Finally, Section 5 concludes this paper.

## 2. Related Work

Existing studies related to this work can be broadly divided into two aspects. The first focuses on deep learning-based defect detection for substation inspection, where generic detection frameworks are adapted to power-equipment inspection tasks. The second focuses on small-object detection and feature enhancement, where multi-scale learning, upsampling enhancement, and attention mechanisms are introduced to improve the representation of subtle defects under complex backgrounds.

### 2.1. Deep Learning-Based Defect Detection for Substation Inspection

Deep learning-based defect detection for substation inspection has evolved rapidly with the development of generic object detection frameworks. In practical inspection applications, one-stage detectors represented by the YOLO family are increasingly preferred because they provide a favorable balance between detection accuracy and inference efficiency, making them suitable for engineering-oriented inspection scenarios.

Recent studies have gradually extended YOLO-based and related deep detection frameworks to power-equipment inspection tasks. For transmission-line inspection, Sadykova et al. [1] applied IN-YOLO to UAV-based outdoor insulator detection, verifying the feasibility of real-time detection in aerial power scenarios. Liu et al. [10] investigated deep-learning-based insulator fault detection using aerial images, further demonstrating the applicability of visual inspection methods to power equipment. For substation inspection scenarios, Zhang et al. [2] introduced image data generation into substation equipment defect detection to alleviate the problem of limited labeled samples. Wang et al. [3] proposed a lightweight detection algorithm for small targets in substation equipment inspection, showing the importance of model efficiency in practical deployment. More recent studies have paid greater attention to defect-oriented feature representation and scene adaptation. Zhang et al. [11] explored adversarial image generation for substation defect samples to enrich training data under limited-sample conditions. Dong et al. [12] developed PHAM-YOLO for substation meter defect detection by introducing a parallel hybrid attention mechanism. Sun et al. [13] improved YOLOv8 for substation equipment defect detection by redesigning the feature extraction structure. Liu et al. [14] proposed a multi-domain collaborative attention fusion model for substation instrument defect detection to improve the identification of subtle surface defects. Luo and Zhang [15] further investigated YOLOX-based defect detection for substations. These studies indicate that substation defect detection is gradually shifting from direct detector application toward data enhancement, architectural redesign, and scene-oriented feature optimization.

Overall, existing studies have demonstrated the effectiveness of deep detectors for substation inspection. However, many methods still concentrate on specific equipment categories or individual defect types. Unified detection for multiple defect classes in complex substation scenes remains insufficient, and the joint problem of subtle defect representation under cluttered backgrounds still requires further investigation.

### 2.2. Small-Object Detection and Feature Enhancement in Substation Inspection

Small-object detection is one of the most critical issues in substation inspection because defect targets are often small, weakly textured, and easily submerged in cluttered backgrounds. Existing substation-oriented studies have attempted to improve such targets through lightweight design, feature enhancement, or attention-based refinement, but these strategies are usually developed for specific equipment or defect categories.

Representative generic feature-learning methods provide important references for this problem. FPN improves cross-scale feature aggregation through a top-down pyramid structure [8], while PANet strengthens bottom-up path aggregation and localization cues [9]. EfficientDet further introduces BiFPN to achieve efficient bidirectional multi-scale feature fusion [16]. These methods show that effective multi-scale aggregation is important for detecting objects with large-scale variation.

In addition to multi-scale aggregation, feature reconstruction and attention mechanisms are also important for weak-target representation. CARAFE improves high-resolution feature reconstruction through content-aware feature reassembly [17], while DySample enhances upsampling by learning dynamic sampling positions [18]. CBAM strengthens feature discrimination through channel and spatial attention [19], and Coordinate Attention embeds positional information into channel attention to improve location-sensitive feature representation [20]. These techniques are particularly relevant to substation inspection, where many defect targets suffer from edge blur, weak local contrast, and incomplete local details under long-distance imaging conditions.

Nevertheless, existing studies mainly focus on multi-scale aggregation, feature reconstruction, or attention refinement independently. The joint problem of preserving shallow fine-grained details during downsampling and adaptively integrating multi-scale features under cluttered backgrounds remains insufficiently explored. This limitation motivates the proposed YOLOv11-SR, which introduces SDPAM and PRGAFM to address these two issues in a unified framework.

## 3. Methods

To address the challenges of explicit fault detection in complex substation inspection scenes, this paper proposes YOLOv11-SR, an improved detection network based on YOLOv11. The proposed method is designed for inspection targets such as meters, respirators, switch cabinets, control boxes, insulators, equipment areas, and ground areas, and aims to detect visible abnormalities including equipment damage, foreign-object attachment, abnormal component states, and safety violations.

**Figure 1:**
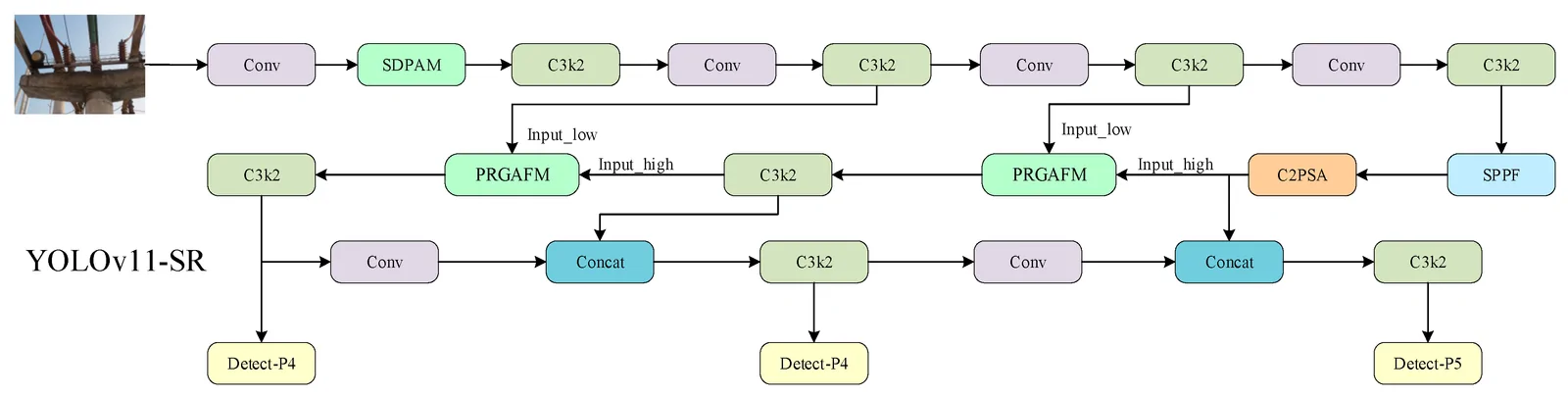
Overall architecture of the proposed YOLOv11-SR network.

As shown in Figure 1, YOLOv11-SR follows the backbone–neck–head architecture of YOLOv11 while introducing two dedicated modules to enhance feature representation and feature fusion. Specifically, a Shallow Detail Preservation Attention Module (SDPAM) is embedded into the shallow downsampling stage of the backbone to alleviate the loss of fine-grained information during early feature extraction and strengthen the preservation of shallow texture, edge, and local structural details. In addition, a Position-Relation Guided Adaptive Fusion Module (PRGAFM) is introduced into the top-down path of the neck to replace the original fixed fusion structure and improve adaptive multi-scale feature interaction under complex backgrounds. Since the detection head and output format remain unchanged, YOLOv11-SR can be trained within the original YOLOv11 detection framework while achieving better detection performance in complex substation scenes.

### 3.1. SDPAM

In substation defect detection, many targets, such as insulator cracks, silica-gel damage, and suspended foreign objects, are usually small in scale, blurred at the boundaries, and easily affected by complex backgrounds. The discrimination of such targets relies heavily on shallow features containing local textures, contours, and structural details. However, in YOLOv11, shallow downsampling is mainly implemented by stride convolution, which can efficiently reduce the feature-map resolution but inevitably causes the loss of fine-grained information, thereby degrading the detection of small and blurred targets. To alleviate this problem, a Shallow Detail Preservation Attention Module (SDPAM) is introduced into the shallow downsampling stage of the backbone to enhance the early representation of small-scale defect targets.

**Figure 2:**
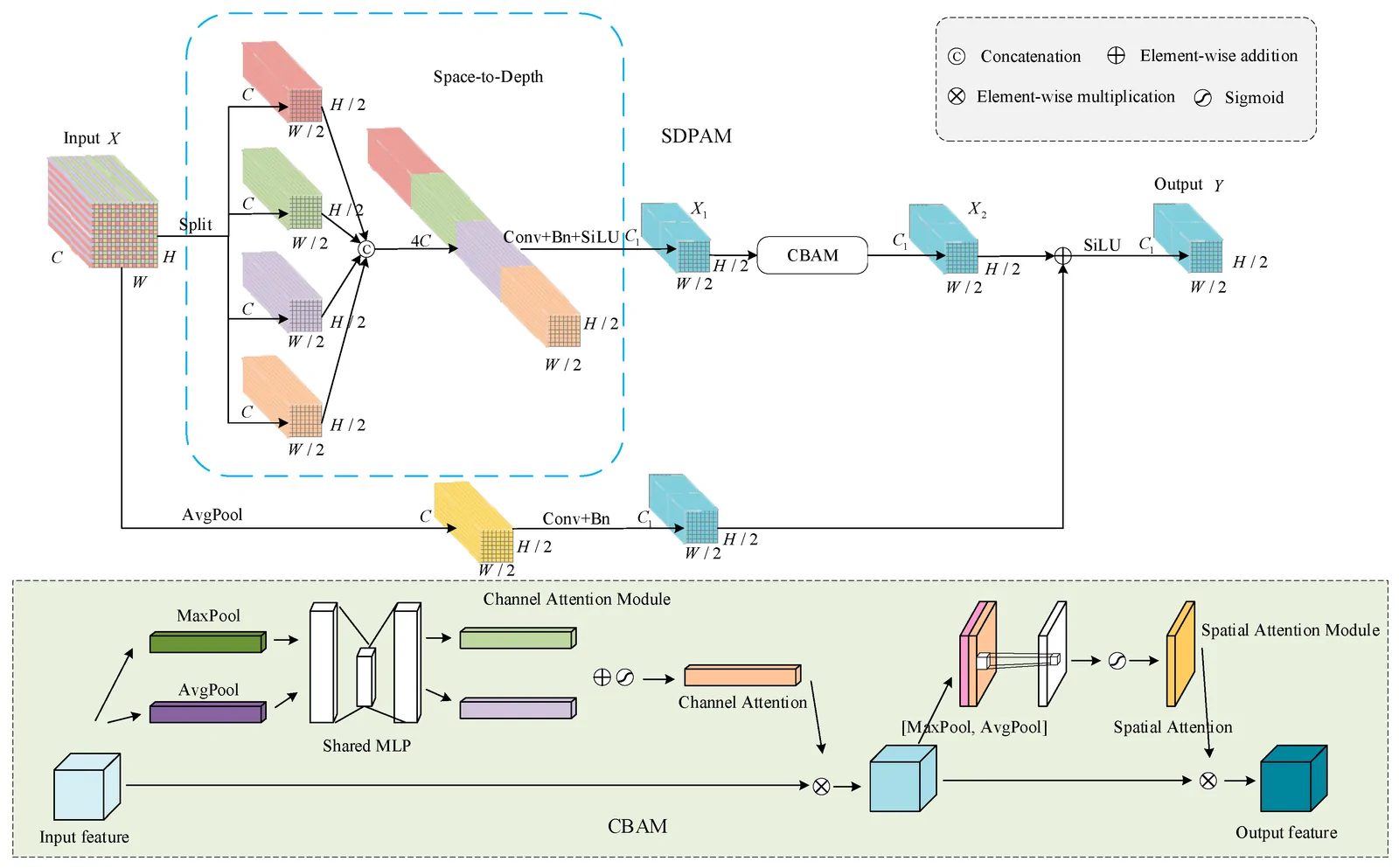
Structure of the shallow detail preservation attention module (SDPAM).

As illustrated in Figure 2, SDPAM consists of a main branch and a residual branch. The main branch sequentially performs Space-to-Depth-based detail-preserving downsampling, local convolutional reconstruction, and CBAM-based attention enhancement. The residual branch is designed to preserve the original shallow information and improve feature propagation through shortcut connections. Let the input feature map be denoted as $X\in {ℜ}^{B\times C\times H\times W}$, where B, C, H and W denote the batch size, channel number, height, and width, respectively. After SDPAM, the output feature map Y is obtained as

(1) $$Y=\delta (CBAM(Conv_{3\times 3}(SPD(X)+R(X))))$$

where SPD(·) denotes the Space-to-Depth operation, Conv(·) denotes a convolutional block composed of convolution, batch normalization, and SiLU activation, CBAM(·) denotes channel-spatial attention enhancement, R(·) denotes the residual branch, and δ(·) is the activation function. As shown in Equation (1), SDPAM integrates shallow downsampling, local feature reconstruction, attention enhancement, and residual compensation into a single feature extraction process.

In the main branch, Space-to-Depth (SPD) is adopted for detail-preserving downsampling. Conventional stride-2 convolution or pooling essentially performs sparse sampling in the spatial domain, which often leads to the loss of local texture and edge information. By contrast, SPD rearranges neighboring spatial features into the channel dimension, thereby reducing spatial resolution while preserving more fine-grained local details. For the input feature $X\in {ℜ}^{B\times C\times H\times W}$, when the block size is set to 2, the dimensional transformation can be expressed as

(2) $$SPD(X)\in {ℜ}^{B\times 4C\times \frac{H}{2}\times \frac{W}{2}}$$

As indicated by Equation (2), after the SPD operation, the spatial resolution is reduced by half, while the number of channels is expanded to four times that of the input feature. In this way, downsampling is achieved without directly discarding local structural information, which is particularly beneficial for detecting subtle cracks, slight damage, and other small targets in substation inspection images.

After the SPD rearrangement, the local information originally distributed across different spatial positions is encoded into the channel dimension. Therefore, a 3×3 convolutional block is introduced into the main branch to perform local modeling and channel compression on the rearranged features, mapping the channel dimension from 4C to the target output channel. Through this process, the local details dispersed in the channel dimension can be reorganized into a more discriminative shallow representation, which provides a stronger basis for subsequent feature enhancement.

On this basis, CBAM is further introduced in SDPAM for feature enhancement. Since substation inspection images usually contain complex backgrounds, such as wires, brackets, equipment textures, and environmental noise, convolutional features alone may still produce insufficient responses to defect targets. CBAM performs joint recalibration in the channel and spatial dimensions, thereby strengthening defect-related salient responses while suppressing background interference. Its operation can be expressed as

(3) $$X_{2}=M_{s}(M_{c}(X_{1})⊗X_{1})⊗M_{c}(X_{1})⊗X_{1}$$

where ⊗ denotes element-wise multiplication, $X_{1}$ and $X_{2}$ denote the input and output features of CBAM, respectively, and $M_{c}(\cdot )$ and $M_{s}(\cdot )$ denote channel attention and spatial attention, respectively. More specifically, the channel and spatial attention maps can be formulated as

(4) $$\left\{\begin{array}{l} M_{c}(X_{1})=\sigma \left(\mathrm{MLP}\left(\mathrm{AvgPool}\left(X_{1}\right)\right)+\mathrm{MLP}\left(\mathrm{MaxPool}\left(X_{1}\right)\right)\right)\\ M_{s}(X_{1})=\sigma \left(f^{7\times 7}\left(\left[\mathrm{AvgPool}\left(X_{1}\right);\;\mathrm{Max}\left(X_{1}\right)\right]\right)\right) \end{array}\right.$$

where σ(⋅) is the Sigmoid function, [⋅ ; ⋅] denotes channel-wise concatenation, and $f^{7\times 7}(\cdot )$ denotes a 7×7 convolution. $\mathrm{AvgPool}\left(\cdot \right)$ and $\mathrm{MaxPool}\left(\cdot \right)$ denote average pooling and max pooling operations, respectively. In channel attention, they are applied to aggregate spatial information, whereas in spatial attention, they are used to aggregate channel information. $\mathrm{MLP}\left(\cdot \right)$ denotes a fully connected layer.

As indicated by Equations (3) and (4), CBAM first emphasizes feature responses that are more relevant to defect discrimination through channel attention, and then further enhances the spatial saliency of potential defect regions through spatial attention. Through this joint recalibration mechanism, SDPAM can more effectively suppress irrelevant interference under complex backgrounds and improve the quality of shallow feature representation, thereby providing a more reliable feature basis for subsequent detection.

The residual branch directly takes the input feature X and performs spatial alignment through average pooling. When the input and output channels are inconsistent, a 1×1 convolution followed by batch normalization is used for channel mapping. The residual output is then added to the enhanced feature from the main branch, and the fused result is passed through the activation function to produce the final output. This design helps stabilize shallow information propagation and improve gradient flow, while avoiding excessive modification of useful original features during the enhancement process.

Overall, SDPAM preserves and strengthens shallow details through four key steps: Space-to-Depth downsampling, local convolutional reconstruction, CBAM-based attention enhancement, and residual compensation. By embedding SDPAM into the shallow downsampling stage of the YOLOv11 backbone, the proposed detector can better represent small-scale defect targets, blurred targets, and distant targets in substation scenes, thereby providing more reliable shallow features for subsequent multi-scale fusion and detection prediction.

### 3.2. PRGAFM

Effective defect detection in substation inspection not only requires strong deep semantic representation but also depends on sufficient utilization of shallow detailed information during multi-scale feature fusion. Although YOLOv11 adopts a multi-scale fusion structure in the neck, its upsampling operation mainly relies on fixed interpolation, and the fusion process is largely based on direct concatenation or feature summation. Such a strategy is efficient but insufficient for modeling the spatially varying contributions of heterogeneous features. To address this issue, a Position-Relation Guided Adaptive Fusion Module (PRGAFM) is introduced into the top-down path of the YOLOv11 neck to replace the original Upsample + Concat + C3k2 structure at the two top-down fusion nodes, thereby improving the selectivity and effectiveness of multi-scale feature interaction.

**Figure 3:**
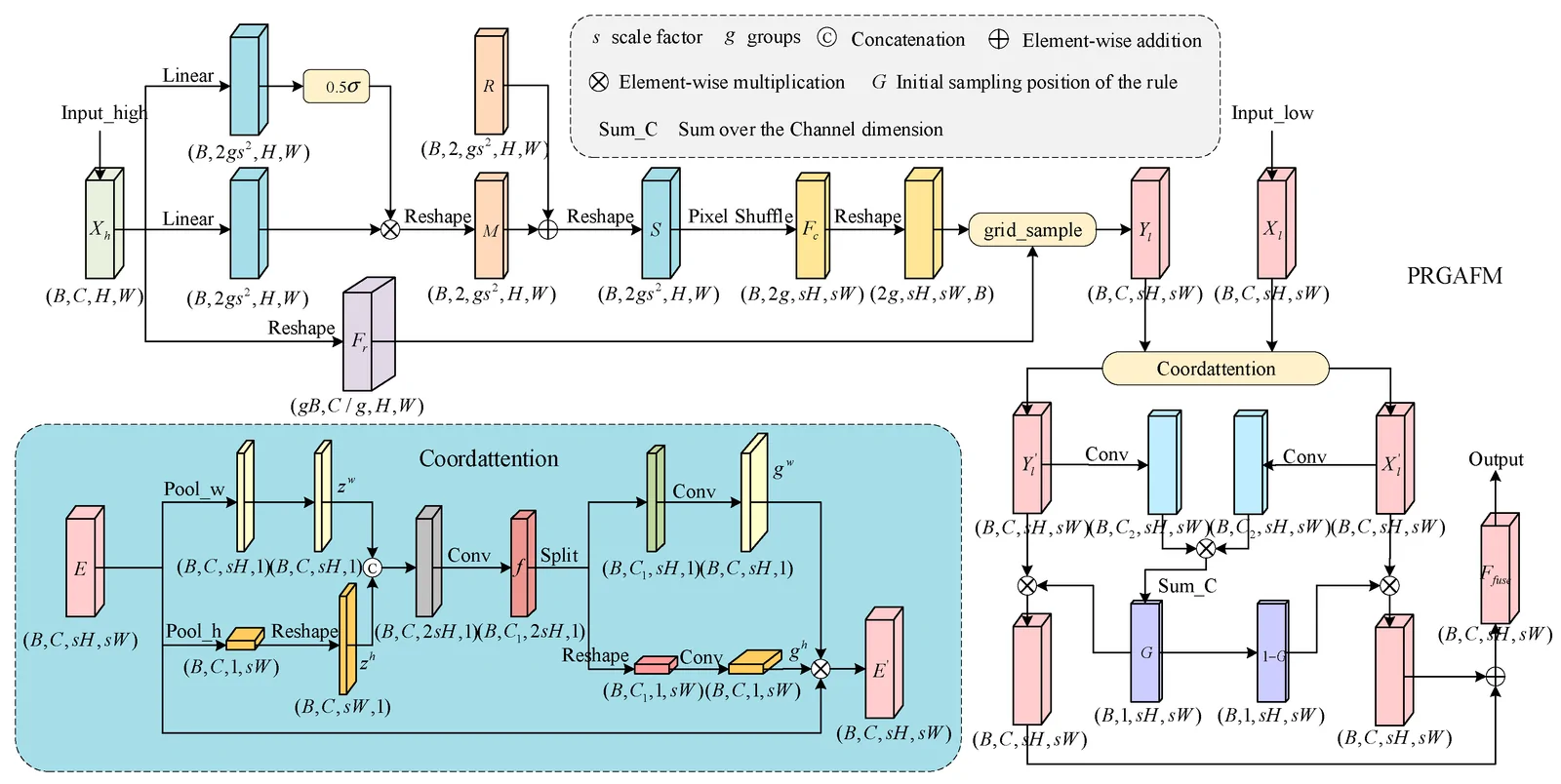
Structure of the position-relation guided adaptive fusion module (PRGAFM).

As illustrated in Figure 3, PRGAFM consists of three main stages: dynamic upsampling, coordinate attention enhancement, and relation-aware gated fusion. Specifically, the module first performs dynamic upsampling on the deep low-resolution feature to recover it to the same spatial scale as the corresponding shallow feature. Then, the deep branch and the shallow branch are aligned in the channel dimension and enhanced separately by coordinate attention. Finally, a relation-aware gating mechanism is used to adaptively fuse the two branches, followed by convolutional refinement to generate the final output feature.

Let the deep and low input features be denoted as $X_{d}$ and $X_{s}$, respectively. Here, $X_{d}$ contains richer semantic information but lower spatial resolution, whereas $X_{s}$ preserves more edge and texture details but is more sensitive to background interference. To improve the interaction between deep semantic features and shallow detailed features, PRGAFM first applies DySample-based dynamic upsampling to the deep feature. Unlike fixed interpolation, DySample predicts learnable sampling offsets and adaptively adjusts the regular sampling grid, making the sampling positions better aligned with target boundaries and local structures. For an input feature $X_{d}\in {ℜ}^{B\times C\times H\times W}$, the module generates the raw offset Δ and dynamic range modulation factor D through two linear projection branches:

(5) $$\left\{\begin{array}{l} \Delta =\mathrm{Linear}\left(X_{d}\right)\\ D=0.5\sigma \left(\mathrm{Linear}\left(X_{d}\right)\right) \end{array}\right.$$

where $\mathrm{Linear}\left(\cdot \right)$ denote linear mappings, Δ and D are of the same spatial size $\left[B,2gs^{2},H,W\right]$, s denotes the upsampling ratio, g denotes the group number. Through these two branches, the module learns the sampling offsets from the input feature while constraining their magnitude using the dynamic range modulation factor, thereby improving the stability of the dynamic sampling process.

The raw offset is then modulated element-wise to obtain the constrained offset $\Delta^{\prime }$, and the final dynamic sampling coordinates are obtained by adding it to the regular base grid $P_{0}$:

(6) $$\left\{\begin{array}{l} \Delta^{\prime }=\Delta ⊗D\\ P=\Delta^{\prime }+P_{0} \end{array}\right.$$

In this way, learnable offsets are superimposed on the regular sampling grid so that the sampling locations can adaptively vary according to the input content.

After obtaining the dynamic sampling coordinates P, the module employs tensor rearrangement and Pixel Shuffle to unfold sub-pixel sampling information from the channel dimension into the high-resolution spatial domain, thereby constructing a dense sampling coordinate map $F_{c}$ consistent with the target output resolution. Meanwhile, the input feature is reorganized into g groups, and the grouped feature is reshaped as $F_{r}$, which not only reduces the computational complexity of the dynamic sampling process but also improves the adaptability of different channel subspaces to local structural variations. Finally, the dynamically upsampled feature is obtained through grid_sample:

(7) $$Y_{s}=\mathrm{grid}\text{\_}\mathrm{sample}(F_{c},F_{r})$$

where $\mathrm{grid}\text{\_}\mathrm{sample}\left(\cdot \right)$ extracts feature values according to the given sampling coordinates and interpolates neighboring values when the sampling locations are non-integer. Therefore, the process in Equation (7) can be regarded as content-adaptive interpolation based on a dynamically generated sampling grid, allowing the recovered deep feature to better align with target boundaries and local structures.

After dynamic upsampling, PRGAFM applies 1×1 convolution to both the deep branch and the shallow branch for channel alignment. Coordinate attention is then applied to the two branches to enhance position-sensitive feature representation. Let the input feature be denoted as $E\in {ℜ}^{B\times C\times H\times W}$, and coordinate attention first performs one-dimensional global aggregation along the width and height directions, respectively:

(8) $$\begin{array}{cc} z_{c}^{h}(h)=\frac{1}{W}\sum_{i=1}^{W}E_{c}(h,i), & z_{c}^{w}(w)=\frac{1}{H}\sum_{j=1}^{H}E_{c}(j,w) \end{array}$$

where $E_{c}(h,i)$ denotes the feature value at channel *c*, row *h*, and column *i*, while $z_{c}^{h}(h)$ and $z_{c}^{w}(w)$ denote the directional descriptors obtained along the width and height directions, respectively. This process decomposes the two-dimensional spatial information into two one-dimensional encodings, thereby preserving positional information while aggregating global context.

The two directional descriptors are then concatenated and passed through a shared transformation to extract a joint representation:

(9) $$f=\delta (Conv_{1\times 1}([z^{h},\;z^{w}]))$$

where [⋅,⋅] denotes concatenation, $Conv_{1\times 1}(\cdot )$ denotes a shared 1 × 1 convolution, and δ(⋅) is a nonlinear activation. The joint representation is then split into two branches to generate the attention weights along the height and width directions:

(10) $$\begin{array}{cc} g^{h}=\sigma (\phi_{h}(f)), & g^{w}=\sigma (\phi_{w}(f)) \end{array}$$

where $\phi_{h}(\cdot )$ and $\phi_{w}(\cdot )$ denote branch-specific transformations, and the final coordinate-attention-enhanced feature is given by

(11) $$E_{c}^{*}=E_{c}⊗g^{h}⊗g^{w}$$

According to Equation (11), coordinate attention jointly modulates the feature through two directional weights. As a result, the deep branch can better highlight semantically salient regions, while the shallow branch can better preserve edge and texture details, thus providing a stronger basis for subsequent adaptive fusion.

After enhancement, the deep branch and the shallow branch produce the refined features $Y_{s}^{*}$ and $X_{s}^{*}$, respectively. PRGAFM then employs a relation-aware gating mechanism to determine whether each spatial location should rely more on deep semantic information or shallow detailed information. Conventional concatenation or summation implicitly assumes that different feature sources are equally important at all spatial locations. However, in real substation scenes, cluttered background regions often require stronger deep semantics to suppress noise, whereas target edges and local defect regions rely more heavily on shallow detailed features to preserve contours and textures. To address this issue, the two branches are first projected into a lower-dimensional space, and a gate map is generated according to their similarity response:

(12) $$G=\sigma (\sum_{c}\left(Conv(X_{s}^{*})⊗Conv(Y_{s}^{*})\right))$$

where $\sum_{c}$ denotes channel-wise summation, and *G* denotes the relation-aware gate map. This gate map reflects the relative contribution and interaction strength between the deep and shallow features at different spatial positions.

According to the gate map *G*, the final fusion result is obtained as

(13) $$F_{fuse}=(1-G)⊗X_{s}^{*}+G⊗Y_{s}^{*}$$

From Equation (13), when the gate value at a given position is small, the fused feature is biased toward the deep branch, thereby emphasizing semantic discrimination. When the gate value is large, the fused feature is biased toward the shallow branch, thereby strengthening edge and texture details. Compared with direct concatenation or fixed-weight fusion, this position-wise adaptive strategy more effectively reduces information redundancy and improves the representation of critical defect regions under complex backgrounds.

### 3.3. Loss Function

Since the proposed modifications are mainly applied to the backbone and neck of YOLOv11, while the detection head and output format remain unchanged, the original YOLOv11 detection loss is directly adopted during training without introducing additional supervision terms. Following the Ultralytics implementation, the total loss consists of three components: the bounding-box regression loss, classification loss, and distribution focal loss, which can be expressed as

(14) $$L=\lambda_{box}L_{box}+\lambda_{cls}L_{cls}+\lambda_{dfl}L_{dfl}$$

where $L_{box}$, $L_{cls}$, and $L_{dfl}$ denote the box loss, classification loss, and distribution focal loss, respectively, and $\lambda_{box}$, $\lambda_{cls}$, and $\lambda_{dfl}$ are the corresponding weighting coefficients. Since the proposed SDPAM and PRGAFM mainly enhance feature extraction and feature fusion without modifying the prediction head, the original YOLOv11 loss remains sufficient for effective training of the improved network. Detailed formulations of each loss term follow the standard Ultralytics YOLOv11 implementation and are therefore omitted here for brevity.

## 4. Experiments

To comprehensively evaluate the proposed YOLOv11-SR for object detection in complex substation scenes, experiments are conducted on a public substation defect dataset. This section presents the dataset description, experimental platform and training settings, comparative results against representative detection networks, category-level performance analysis, and ablation studies on the proposed modules. The evaluation focuses on detection accuracy, model complexity, and inference speed, and is further supported by visualization examples.

### 4.1. Dataset

**Figure 4:**
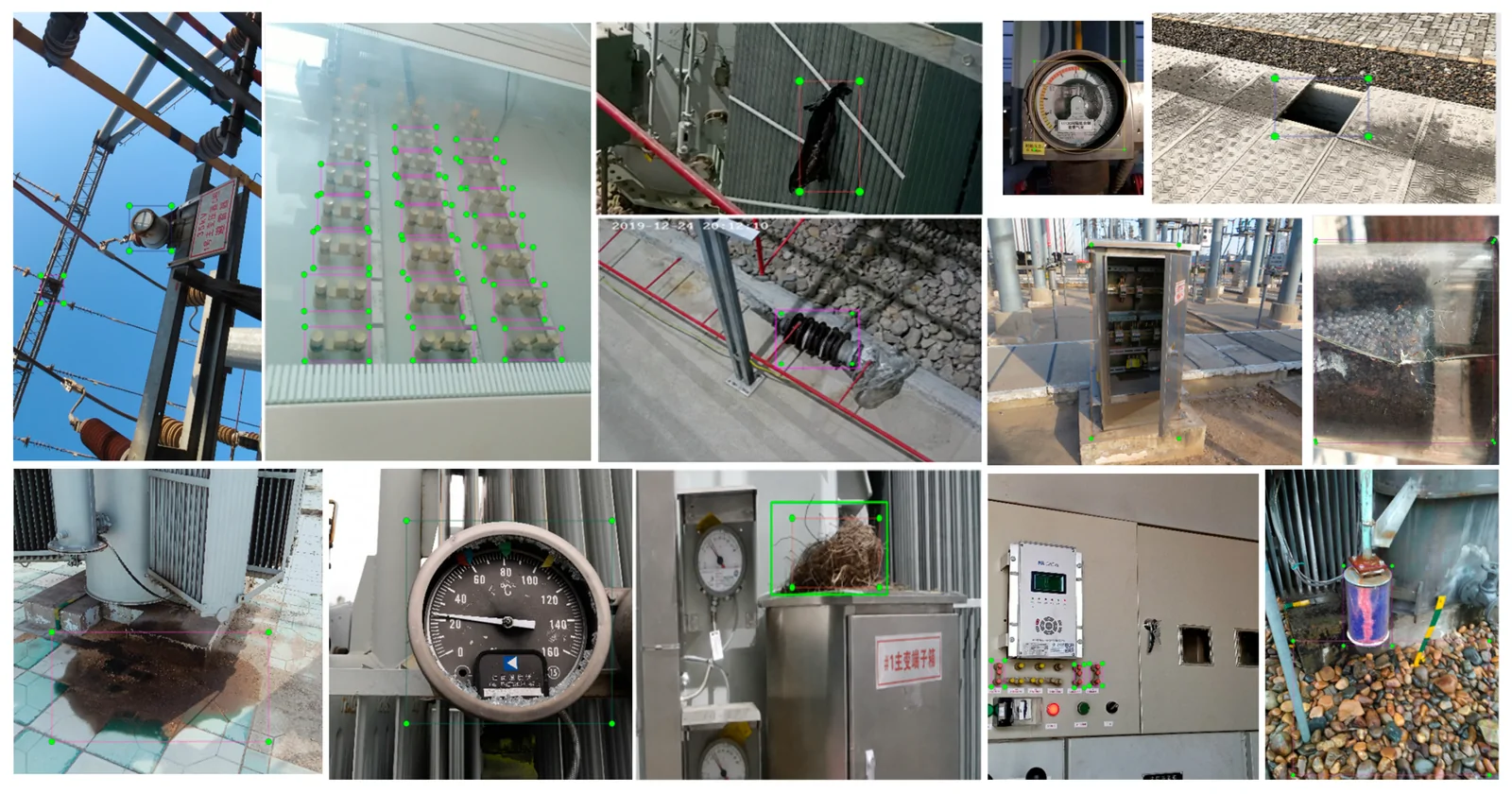
Examples of explicit fault targets in substation inspection scenes.

The dataset used in this work is derived from a public substation inspection dataset (https://blog.csdn.net/FL1623863129/article/details/139365811 (accessed 7 March 2026)) for intelligent detection of equipment defects and abnormal conditions in substation scenarios. It contains 6588 images collected from 7 substation scenarios and covers typical inspection targets such as meters, respirators, switch cabinets, control boxes, insulators, equipment areas, and ground areas, involving 13 defect or status categories in total. To clearly describe the correspondence between inspection targets, defect categories, and label IDs, the dataset is organized in the form of “inspection object—defect category—label ID,” as summarized in Table 1. Some representative defect scenes are shown in Figure 4.

### 4.2. Experimental Platform and Training Settings

All experiments are conducted on a workstation running Windows 11 Professional, with PyCharm 2023.1 as the development environment. Model training and testing are implemented in Python 3.8 using PyTorch 2.3.0, with CUDA 12.1 for hardware acceleration. The platform is equipped with an Intel^®^ Core™ i7-14700K CPU and an NVIDIA GeForce RTX 4070 Ti SUPER GPU.

The dataset is divided into 3953 training images, 1379 validation images, and 1256 test images. The proposed model is trained based on the YOLOv11 detection framework. The input image size is set to 640 × 640, the number of training epochs is 200, and the batch size is 16. The optimizer is stochastic gradient descent (SGD), with an initial learning rate of 0.01, a final learning-rate factor of 0.1, a momentum of 0.937, and a weight decay of 0.0005. To improve training stability at the early stage, the number of warm-up epochs is set to 3, while the warm-up momentum and bias learning rate are set to 0.8 and 0.1, respectively.

For data augmentation, HSV color perturbation, random translation, random scaling, horizontal flipping, Mosaic, and MixUp are adopted to improve the robustness of the detector to complex-scene samples. To ensure fair comparison, all models share the same hardware environment, dataset split, input resolution, training schedule, optimizer setting, and data augmentation strategy, differing only in network architecture.

### 4.3. Evaluation Metrics

To comprehensively evaluate the compared models, macro-averaged Precision (P), Recall (R), F1-score, together with mAP@0.5 and mAP@0.5:0.95, are adopted as the main detection metrics, while the number of parameters, GFLOPs, and frames per second (FPS) are used to assess model complexity and inference efficiency. The reported Precision, Recall, and F1-score are calculated using the macro-average over all 13 defect categories, where the metric is first computed for each category and then averaged across categories. Precision represents the proportion of correctly detected positive samples among all predicted positive samples, whereas Recall measures the proportion of correctly detected positive samples among all ground-truth positive samples. The F1-score is the harmonic mean of precision and recall. Precision, recall, and F1-score are calculated as follows:

(15) $$P=\frac{TP}{TP+FP},\;R=\frac{TP}{TP+FN},\;F1=\frac{2PR}{P+R}$$

where TP, FP, and FN denote the numbers of true-positive, false-positive, and false-negative detections, respectively.

The intersection over union (IoU) measures the overlap between the predicted bounding box *B_p_* and the corresponding ground-truth bounding box *B_g_*, and is defined as

(16) $$\mathrm{IoU}=\frac{\mathrm{Area}(B_{p}\cap B_{g})}{\mathrm{Area}(B_{p}\cup B_{g})}$$

The mean average precision (mAP) is calculated by averaging the average precision values over all categories:

(17) $$\mathrm{mAP}=\frac{1}{C}\sum_{c=1}^{C}AP_{c}$$

where $AP_{c}$ denotes average precision (AP) of the *c*-th category, and C denotes the total number of categories. The AP is calculated as the area under the precision–recall curve:

(18) $$\mathrm{AP}=\int_{0}^{1}P(R)dR$$

where P(R) represents the precision value corresponding to a given recall value. The mAP@0.5 metric is calculated at an IoU threshold of 0.5, whereas mAP@0.5:0.95 is obtained by averaging the mAP values over IoU thresholds ranging from 0.5 to 0.95 with an interval of 0.05. Compared with mAP@0.5, mAP@0.5:0.95 imposes stricter localization requirements and therefore provides a more comprehensive evaluation of detection performance.

### 4.4. Comparative Analysis with Different Networks

**Figure 5:**
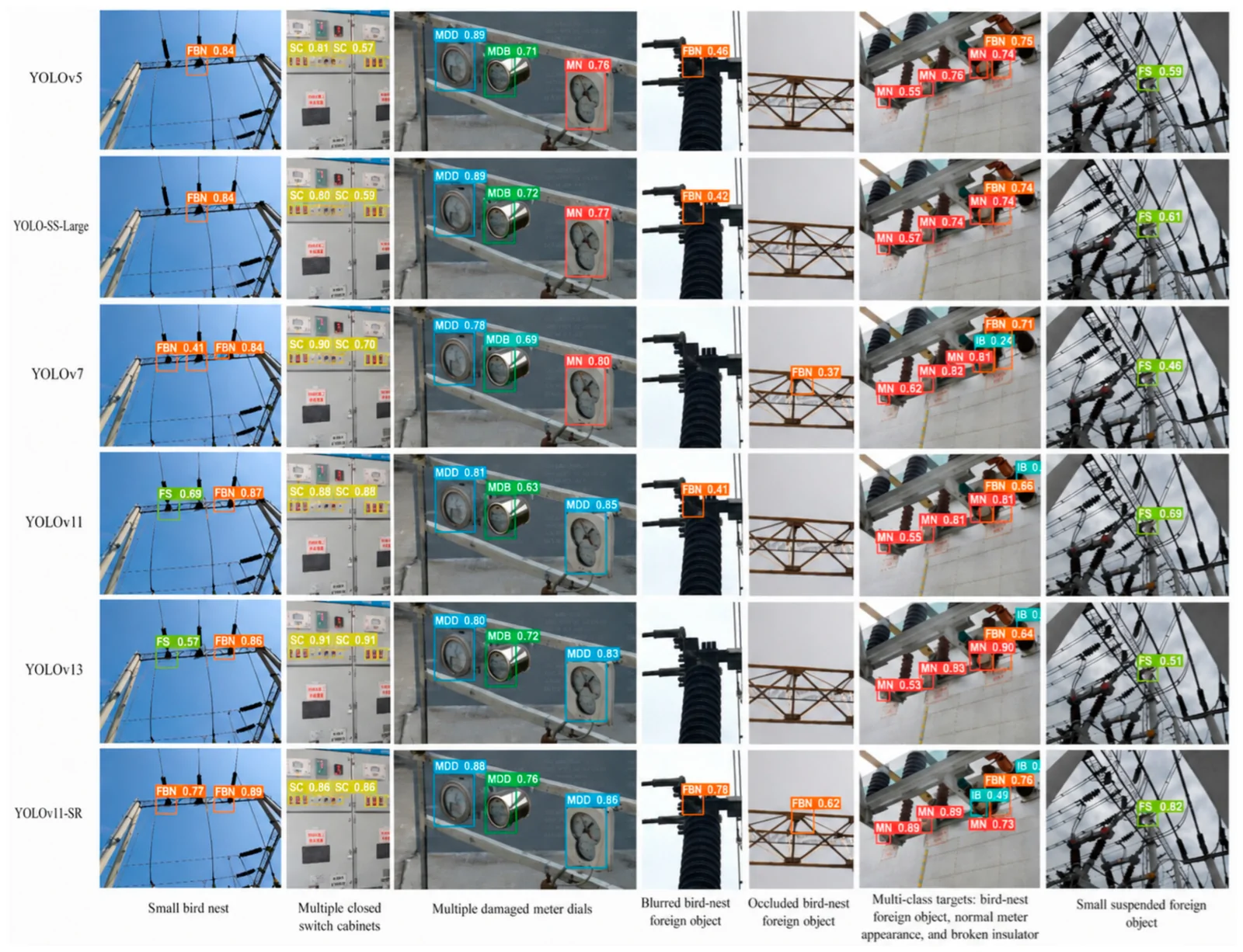
Examples of defect categories in the substation inspection dataset. Category abbreviations used in this figure are defined in Table 1.

To evaluate the effectiveness of the proposed YOLOv11-SR, several representative detection networks are selected for comparison, including YOLOv5, YOLOv7 [5], YOLOv11 [6], YOLOv13 [7], and YOLO-SS-Large [21]. The quantitative and qualitative results are listed in Table 2 and Figure 5, respectively.

From the quantitative perspective, YOLOv11-SR achieves 89.9% Precision, 87.8% Recall, 89.0% F1-score, 90.7% mAP@0.5, and 64.8% mAP@0.5:0.95. Compared with the other detectors, YOLOv5 and YOLOv7 show relatively weaker overall performance, indicating limited feature representation and localization capability in complex scenes. YOLO-SS-Large exhibits clear advantages in model compactness, computational complexity, and inference speed, with only 12.4 M parameters and 28.3 GFLOPs, but its detection accuracy is significantly lower than that of YOLOv11-SR, suggesting that the lightweight design sacrifices discriminative capability in challenging substation scenarios. YOLOv13 outperforms YOLOv11 on some metrics, but its overall performance remains inferior to YOLOv11-SR. Meanwhile, YOLOv11-SR contains 54.6 M parameters and 233.0 GFLOPs, compared with 52.7 M parameters and 181.2 GFLOPs for YOLOv11, while maintaining 58.47 FPS. These results indicate that the proposed method significantly improves detection accuracy with only a modest increase in model complexity while preserving satisfactory inference efficiency. Overall, YOLOv11-SR achieves the best performance on all major metrics, including Precision, Recall, F1-score, mAP@0.5, and mAP@0.5:0.95, demonstrating stronger overall detection capability in complex substation scenes.

From the qualitative perspective, as shown in Figure 5, YOLOv11-SR exhibits better detection stability in scenarios involving small objects, blurred objects, multiple objects, occlusion, and cluttered backgrounds. Its main advantages are reflected in fewer missed detections, fewer misclassifications, and higher confidence scores, indicating stronger adaptability to complex substation inspection conditions.

### 4.5. Category-Level Performance Analysis

To further examine the category-wise detection performance of the improved network, the AP@0.5 and AP@0.5:0.95 results of the baseline YOLOv11 and the improved YOLOv11-SR on all 13 categories are reported in Table 3. Overall, the improved network achieves gains on most categories, with average improvements of 4.8 and 5.5 percentage points in AP@0.5 and AP@0.5:0.95, respectively. These results indicate that the proposed network provides more stable category recognition and object localization performance in complex inspection scenes.

More noticeable improvements are observed in categories such as bird nest, suspended foreign object, broken insulator, blurred meter dial, and respirator silica-gel damage. These categories are typically characterized by small target size, blurred boundaries, weak local structures, or strong background interference, and are therefore more difficult to detect. In particular, respirator silica-gel damage contains only 47 samples and is a typical difficult category with limited training data. The clear improvement on such categories indicates that the proposed network is more effective for challenging cases. By contrast, the performance gap is relatively small for categories such as respirator silica-gel discoloration and ground oil leakage, mainly because these targets have more salient appearance features and are easier for the baseline to detect. Overall, the advantage of YOLOv11-SR is more evident on difficult categories and complex-scene categories.

### 4.6. Effectiveness Analysis of PRGAFM

To further validate the effectiveness of PRGAFM, a comparison with representative feature fusion strategies, including BiFPN and ASFF, is conducted under the same experimental settings. Specifically, these feature fusion mechanisms are integrated into the neck of YOLOv11 while keeping the backbone, detection head, training strategy, and dataset unchanged. The quantitative results are summarized in Table 4.

As shown in Table 4, both BiFPN and ASFF improve the baseline performance, whereas PRGAFM achieves the best overall results. BiFPN strengthens multi-scale interaction through weighted bidirectional aggregation, while ASFF adaptively selects spatial features across different scales. By jointly exploiting positional information and inter-feature relationships, PRGAFM provides more effective cross-scale feature fusion, particularly for small, blurred, and partially occluded targets. Compared with ASFF, PRGAFM improves Precision, Recall, F1-score, mAP@0.5, and mAP@0.5:0.95 while maintaining comparable model complexity and inference speed. These results demonstrate that the proposed PRGAFM can achieve more effective feature fusion without introducing a noticeable increase in computational cost.

### 4.7. Ablation Analysis

To verify the effectiveness of the proposed modules, ablation experiments are conducted on the baseline network by separately introducing SDPAM, PRGAFM, and their combination. The results are reported in Table 5. Overall, both SDPAM and PRGAFM individually improve the baseline performance, and the combination of the two modules yields the best results, indicating that they are complementary for object detection in complex scenes.

From the quantitative perspective, introducing SDPAM improves Precision, Recall, and F1-score to 85.9%, 84.7%, and 84.7%, respectively, while mAP@0.5 and mAP@0.5:0.95 increase to 87.3% and 61.9%. These results indicate that SDPAM effectively enhances shallow-detail representation. When PRGAFM is introduced, the model achieves 85.3% Precision, 83.1% Recall, 84.3% F1-score, 88.0% mAP@0.5, and 62.6% mAP@0.5:0.95, showing that this module effectively improves feature fusion quality under cluttered backgrounds. When both modules are used together, the model achieves the best overall performance with 54.6 M parameters, 233.0 GFLOPs, and an inference speed of 58.47 FPS. These results further confirm the effectiveness and rationality of the proposed design.

**Figure 6:**
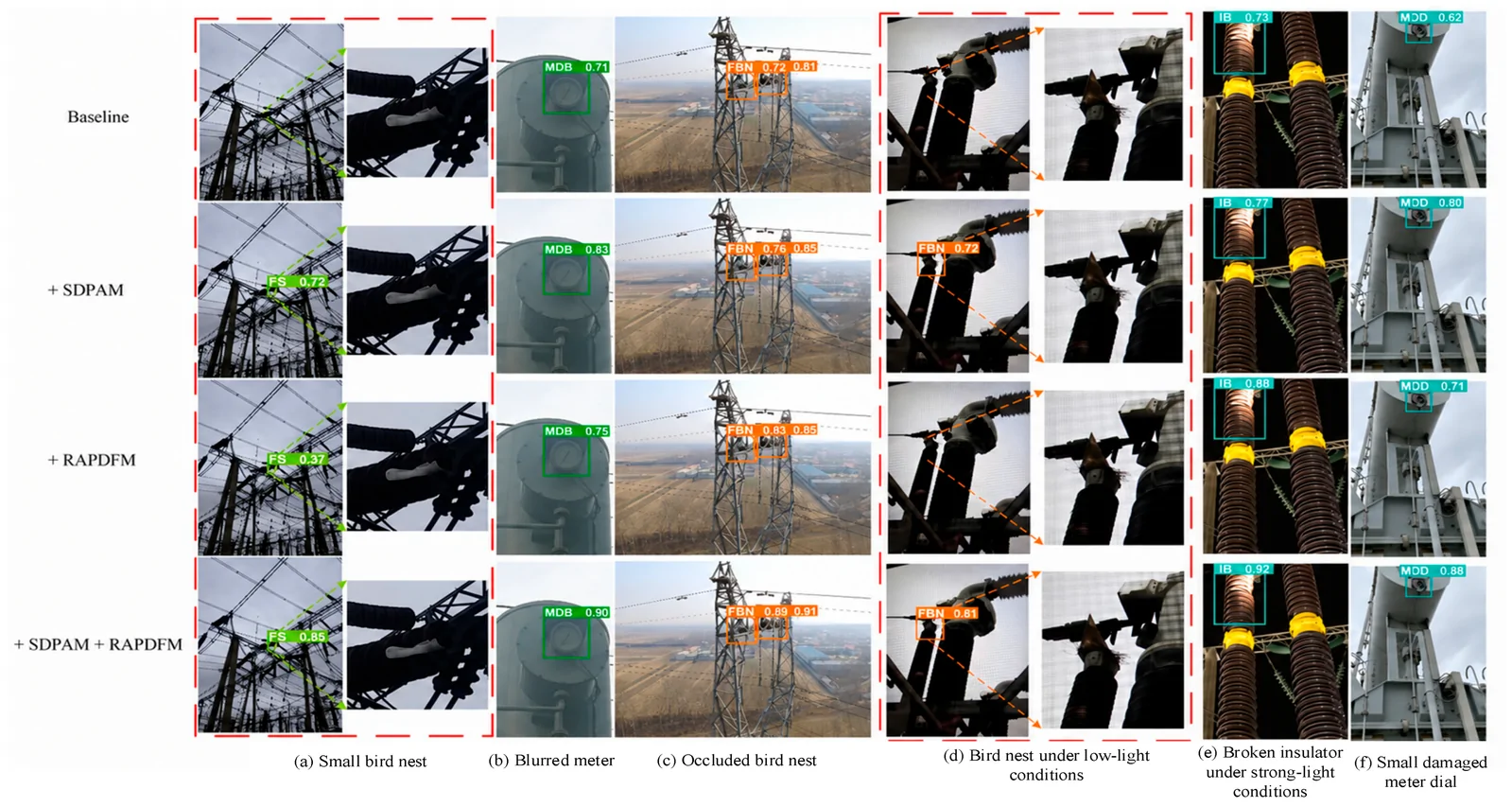
Visualization results of the ablation study. Category abbreviations used in this figure are defined in Table 1.

From the qualitative perspective, as shown in Figure 6, the joint model generally produces the highest confidence and the most stable predictions in challenging scenarios such as small objects, blurred objects, partial occlusion, and low illumination. Overall, SDPAM is more beneficial for enhancing detailed target representation, whereas PRGAFM contributes more to stable feature fusion in complex scenes. Their combination yields the best detection performance, which is consistent with the quantitative ablation results.

## 5. Conclusions

This paper proposes YOLOv11-SR for explicit fault detection in complex substation inspection scenes. By introducing the Shallow Detail Preservation Attention Module (SDPAM) and the Position-Relation Guided Adaptive Fusion Module (PRGAFM), the proposed method strengthens shallow detail representation and improves multi-scale feature fusion under cluttered backgrounds, thereby enhancing the detection of small and blurred targets. Experimental results show that YOLOv11-SR achieves superior overall performance on the public substation defect dataset, particularly for challenging cases involving small objects, blurred boundaries, weak local structures, and complex background interference. Category-level analysis further confirms its effectiveness on difficult defect categories, while ablation studies verify the individual and complementary contributions of SDPAM and PRGAFM.

Despite these improvements, the proposed method still introduces a modest computational overhead and has only been evaluated on a single public substation dataset using RGB images. Future work will focus on lightweight model design, cross-dataset and cross-substation validation, and multimodal information fusion to further improve deployment efficiency and generalization capability.

## Figures and Tables

**Table 1:** Defect categories and label definitions in the substation inspection dataset.

Object	Defect Category	Label ID	Samples
Meter	Normal meter appearance; Meter shell damage; Blurred meter dial; Broken meter dial	meter_normal (MN); meter_shell_damage (MSD); meter_dial_blur (MDB); meter_dial_damage (MDD)	789; 523; 869; 723
Respirator	Silica-gel discoloration; Silica-gel damage	resp_silica_discolor (RSDC); resp_silica_damage (RSD)	1174; 106
Switch Cabinet	Switch cabinet closed	switch_closed (SC)	362
Control Box	Abnormal control-box door closure	Ctrlbox_door_abnormal (CDA)	383
Insulator	Broken insulator	insulator_breakage (IB)	410
Equipment Area	Suspended foreign object; Bird nest	foreign_suspended (FS); foreign_bird_nest (FBN)	729; 883
Ground Area	Cover plate damage; Ground oil leakage	cover_damage (CD); ground_oil_leakage (GOL)	654; 833

**Table 2:** Performance comparison of different detection networks.

Networks	P (%)	R (%)	F1 (%)	mAP@0.5 (%)	mAP@0.5:0.95 (%)	Parameters (M)	GFLOPs	FPS
YOLOv5	79.6	70.8	74.5	75.9	47.3	46.5	109.1	37.56
YOLOv7	78.9	72.5	75.6	78.2	51.8	36.6	103.5	64.85
YOLOv11	82.5	79.6	81.4	85.9	59.3	52.7	181.2	67.38
YOLOv13	84.1	78.1	81.6	83.1	61.1	51.8	162.1	60.09
YOLO-SS- Large	79.4	70.4	74.6	76.4	47.2	12.4	28.3	173.62
YOLOv11-SR	89.9	87.8	89.0	90.7	64.8	54.6	233.0	58.47

Note: P, R, and F1 are macro-averaged over all 13 categories.

**Table 3:** Category-level detection performance of YOLOv11 and YOLOv11-SR.

Category	Samples	YOLOv11		YOLOv11-SR	
		AP@0.5/%	AP@0.5:0.95%	AP@0.5/%	AP@0.5:0.95/%
meter_normal	298	87.2	61.8	91.4	66.5
meter_shell_damage	102	84.9	58.0	89.3	63.2
meter_dial_blur	146	85.6	59.2	91.0	65.5
meter_dial_damage	220	89.2	63.1	93.0	67.9
resp_silica_discolor	267	95.2	72.0	96.5	74.8
resp_silica_damage	47	75.6	41.8	85.2	51.8
switch_closed	162	87.6	63.7	90.3	66.8
ctrlbox_door_abnormal	83	81.2	53.2	87.2	60.1
insulator_breakage	264	82.2	54.0	88.2	61.0
foreign_suspended	386	84.0	56.1	90.0	63.0
foreign_bird_nest	200	82.6	54.9	91.0	63.2
cover_damage	117	89.7	66.3	92.5	69.2
ground_oil_leakage	200	91.4	67.1	93.6	70.0
Average	192	85.9	59.3	90.7	64.8

**Table 4:** Comparison of different feature fusion strategies.

Networks	P (%)	R (%)	F1 (%)	mAP@0.5 (%)	mAP@0.5:0.95 (%)	Parameters (M)	GFLOPs	FPS
YOLOv11	82.5	79.6	81.4	85.9	59.3	52.7	181.2	67.38
+BiFPN	83.6	83.7	82.3	86.5	61.4	49.1	172.1	70.33
+ASFF	83.1	83.8	81.9	86.2	60.7	54.6	186.6	67.14
+PRGAFM	85.3	83.1	84.3	88.0	62.6	53.9	187.1	67.12

**Table 5:** Ablation study of the proposed modules.

Networks	P (%)	R (%)	F1 (%)	mAP@0.5 (%)	mAP@0.5:0.95 (%)	Parameters (M)	GFLOPs	FPS
Baseline	82.5	79.6	81.4	85.9	59.3	52.7	181.2	67.38
+SDPAM	85.9	84.7	84.7	87.3	61.9	53.6	226.9	65.17
+PRGAFM	85.3	83.1	84.3	88.0	62.6	53.9	187.1	67.12
+SDPAM + PRGAFM	89.9	87.8	89.0	90.7	64.8	54.6	233.0	58.47

## Data Availability

The data that support this study are freely available and were downloaded from the following public domain resources: [https://blog.csdn.net/FL1623863129/article/details/139365811 (accessed 7 March 2026)]. All data are available upon request from the corresponding author.
